# Dietary Fibre Intake Is Associated with Serum Levels of Uraemic Toxins in Children with Chronic Kidney Disease

**DOI:** 10.3390/toxins13030225

**Published:** 2021-03-19

**Authors:** Amina El Amouri, Evelien Snauwaert, Aurélie Foulon, Charlotte Vande Moortel, Maria Van Dyck, Koen Van Hoeck, Nathalie Godefroid, Griet Glorieux, Wim Van Biesen, Johan Vande Walle, Ann Raes, Sunny Eloot

**Affiliations:** 1 Paediatric Nephrology and Rheumatology Section, Department of Paediatrics, Ghent University Hospital, Corneel Heymanslaan 10, 9000 Ghent, Belgium; Evelien.Snauwaert@UZGent.Be (E.S.); Johan.VandeWalle@uzgent.be (J.V.W.); ann.raes@ugent.be (A.R.); 2 Nephrology Section, Department of Internal Medicine and Paediatrics, Ghent University Hospital, Corneel Heymanslaan 10, 9000 Ghent, Belgium; aurelie.foulon@telenet.be (A.F.); charlotte.vandemoortel@ugent.be (C.V.M.); Griet.Glorieux@UGent.be (G.G.); wim.vanbiesen@uzgent.be (W.V.B.); Sunny.Eloot@UGent.be (S.E.); 3 Paediatric Nephrology Section, Department of Paediatrics, University Hospitals Leuven (Campus Gasthuisberg), Herestraat 49, 3000 Leuven, Belgium; maria.vandyck@uzleuven.be; 4 Paediatric Nephrology Section, Department of Paediatrics, Antwerp University Hospital, Wilrijkstraat 10, 2650 Edegem, Belgium; koen.van.hoeck@uza.be; 5 Paediatric Nephrology Section, Department of Paediatrics, Cliniques Universitaires St. Luc, Université Catholique Louvain, Avenue Hippocrate 10, 1200 Brussels, Belgium; nathalie.godefroid@uclouvain.be

**Keywords:** chronic kidney disease, children, diet, fibre intake, uraemic toxins

## Abstract

Imbalanced colonic microbial metabolism plays a pivotal role in generating protein-bound uraemic toxins (PBUTs), which accumulate with deteriorating kidney function and contribute to the uraemic burden of children with chronic kidney disease (CKD). Dietary choices impact the gut microbiome and metabolism. The aim of this study was to investigate the relation between dietary fibre and gut-derived PBUTs in paediatric CKD. Sixty-one (44 male) CKD children (9 ± 5 years) were prospectively followed for two years. Dietary fibre intake was evaluated by either 24-h recalls (73%) or 3-day food records (27%) at the same time of blood sampling for assessment of total and free serum levels of different PBUTs using liquid chromatography. We used linear mixed models to assess associations between fibre intake and PBUT levels. We found an inverse association between increase in fibre consumption (g/day) and serum concentrations of free indoxyl sulfate (−3.1% (−5.9%; −0.3%) (*p* = 0.035)), free p-cresyl sulfate (−2.5% (−4.7%; −0.3%) (*p* = 0.034)), total indole acetic acid (IAA) (−1.6% (−3.0%; −0.3%) (*p* = 0.020)), free IAA (−6.6% (−9.3%; −3.7%) (*p* < 0.001)), total serum p-cresyl glucuronide (pCG) (−3.0% (−5.6%; −0.5%) (*p* = 0.021)) and free pCG levels (−3.3% (−5.8%; −0.8%) (*p* = 0.010)). The observed associations between dietary fibre intake and the investigated PBUTs highlight potential benefits of fibre intake for the paediatric CKD population. The present observational findings should inform and guide adaptations of dietary prescriptions in children with CKD.

## 1. Introduction

Chronic kidney disease (CKD) in children is a micro-inflammatory state, affecting nearly every organ system and resulting into an increased morbidity and high mortality, along with a decreased quality of life [1,2,3,4]. The exact pathophysiological mechanisms underlying the complex and multifactorial paediatric uraemic syndrome are still poorly understood. In recent years, it was postulated that the accumulation of organic waste products with failing kidney function, is one of the key contributors of uraemic illness [5]. A large number of these uraemic toxins arise from protein fermentation by the gut microbiota and circulate in the blood bound to albumin [5,6,7]. Two of the most studied protein-bound uraemic toxins (PBUTs), indoxyl sulfate (IxS) and p-cresyl sulfate (pCS), might be promising targets for adjuvant toxin-reductive strategies [8]. Accruing observational and experimental studies suggest that these two toxins, as well as the less studied p-cresyl glucuronide (pCG) and indole acetic acid (IAA), are associated with poor cardiovascular outcomes and kidney disease progression [8,9,10,11,12].

The link between the gut and the kidney seems to be bidirectional, since uraemic patients also seem to have a unique dysbiotic colon microenvironment with profoundly altered composition and metabolism of gut microbes and prolonged transit time [8,13,14,15,16]. As the intestinal microbial metabolism is largely driven by nutrient availability, it was postulated that dietary interventions could reduce toxin generation [16,17]. A higher intake of dietary fibre may be a suitable candidate to restore the balance, since it reduces the transit time, might promote beneficial microbial species, and shifts a dominantly proteolytic microbial metabolism to a saccharolytic one. In saccharolysis, amino acids are incorporated for bacterial growth and used as an energy source, rather than being metabolised in precursors of uraemic solutes [16,18]. So far, supplementation studies under uraemic conditions in humans have been scarce and unsatisfactory. Meta-analyses underline the weak/suboptimal body of evidence, mainly due to significant study heterogeneity (study population, methodology, duration) and selection of unsuitable fibre types, while dietary assessments are very rarely formally performed [17,19,20,21,22]. Moreover, most patients with CKD are on a diet restricting the intake of fibre-rich fruits and vegetables because of their potassium content. Furthermore, low fibre intake was associated with a higher risk of inflammation and mortality in adult CKD patients [23]. Recent studies demonstrated that fibre intake is inadequate in adult as well as paediatric patients with CKD. Daily fibre intake in these patients is far below the recommendations for the healthy population and inversely related to advancing CKD stages [24,25].

In our recently published observational study, we found that patients with the highest fibre intake had lower total and free pCG levels. This study was a cross-sectional ana-lysis, therefore the confounding effect of intra-patient variability in dietary intake and uraemic toxin concentrations could not be excluded. A study in an adult haemodialysis population found a marked intra-patient variability, possibly affecting the significance of the associations between a single concentration of certain toxins (especially total IxS), and outcomes [26]. Awaiting similar trials to determine whether these conclusions are applicable to a paediatric population with varying degrees of kidney dysfunction, we aimed to take into account potential fluctuations within one patient over time, by conducting longitudinal mixed-model analyses, using serial PBUT concentration measurements in blood samples taken at the same time of repeated dietary intake evaluations.

Therefore, the purpose of this study was to evaluate the longitudinal association between dietary fibre intake and total and free serum levels of four selected PBUTs: IxS, pCS, IAA and pCG.

## 2. Results

A total of 61 children (9 ± 5 years) were eligible for analysis, accounting for a total of 297 visits, with a mean number of 5 visits/patient (range 1–9) and a median follow-up time of 19 (9–22) months. Baseline characteristics are listed in Table 1. Drop out of patients was attributed to dialysis initiation (n = 6) or kidney transplantation (n = 2). Other missing data originated from missing dietary records or the lack of appropriate coupling of dietary intake with serum PBUT levels the same day. In this CKD cohort, 33% of the children was diagnosed with CKD stage 1–2, 31% with stage 3, 28% with stage 4 and 8% with stage 5. Dietary assessment was obtained through 3-day food records in 27% of the visits and through 24-h recalls in 73% of the visits. On average, only 76% of the Dietary Reference Intake (DRI) for fibre was achieved, while only 23% of children reached 100% of the DRI. In contrast, 92% of children achieved the 100%DRI for protein. Nutrient intake and PBUT levels across different stages of CKD can be found in Supplementary Tables S1 and S2.

As shown in Table 2, after adjustment for body surface area (BSA), estimated glomerular filtration rate (eGFR) and protein intake, for every g/day increase in fibre consumption, mixed-model analysis revealed a 1.6% (−3.0%; −0.3%) lower total IAA concentration (*p* = 0.020), whereas free IAA levels were 6.6% (−9.3%; −3.7%) (*p* < 0.001) lower. Further, total pCG levels were 3.0% (−5.6%; −0.5%) (*p* = 0.021) lower, and free serum pCG 3.3% (−5.8%; −0.8%) (*p* = 0.010) lower per g/day increase in daily fibre consumption.

For every gram of increment in daily fibre intake, free IxS levels were 3.1% (−5.9%; −0.3%) (*p* = 0.035) lower, and free pCS were 2.5% (−4.7%; −0.3%) (*p* = 0.034) lower. In contrast, total IxS and pCS serum concentrations were not associated with daily fibre intake (Table 2).

## 3. Discussion

This longitudinal, observational study investigated whether dietary fibre intake is linked to levels of circulating PBUTs in a paediatric CKD cohort. We showed that independent of eGFR, each g/day fibre intake increase was associated with dose-dependent lower levels of free pCS and IxS and both free and total pCG and IAA concentrations.

Our findings are in line with a study in adult patients with CKD stage 3–4 which found significantly lower serum IxS levels in a high-fibre versus a low-fibre intake group [27]. Another observational cohort study in adult non-dialysed patients with CKD 4–5 found that a high dietary fibre intake was negatively correlated with both total and free pCS, but not with IxS [28]. To our knowledge, there are no interventional trials supplementing fibre prebiotics in the paediatric CKD population. In adult haemodialysis (HD) patients, beneficial effects of prebiotics on either pCS [29] or (free) IxS [18,30] have been demonstrated. With the exception of one single-blind pilot study, which found a lower level of p-cresol after combined pea hull fiber and inulin supplementation [31], three other trials performed in adult CKD patients who were not on dialysis found no effect on either pCS or IxS after administration of different prebiotics [32,33,34]. The only two studies evaluating either pCG or IAA in adults with CKD found no significant reduction [32,34]. The essentially negative results in adult patients with CKD who were not on dialysis might be attributable to the aforementioned heterogeneity in study design and fibre choice. In addition, dietary intake was often not taken into account in these analyses.

Our data suggest that fibre intake might primarily influence free PBUT concentrations. It is hypothesised that changes in free solute levels of PBUTs are more relevant than total concentrations in the pathophysiology of the uraemic syndrome [18,35]. In analogy with studies of protein-bound pharmaceutical agents, the free fraction is regarded as the biologically active one and is considered a better indicator of potential toxicity [17,18,35,36,37]. Several studies investigating pCS concentrations in adult patients either with CKD or on haemodialysis revealed that only the free levels were associated with cardiovascular morbidity and mortality [38,39,40]. Although still under discussion, it has therefore been suggested that future studies exploring clinically relevant markers and outcomes should take into account both total and free concentrations [35].

Nutrient availability, more specifically the balance between undigested protein and carbohydrate, can modulate microbial metabolism towards either saccharolytic or proteolytic fermentation [16]. The protein/fibre index, taking into account the interplay between the two single nutrients, could thus be informative about the prevailing fermentation profile. In our study, 92% of the children achieved the daily recommended protein intake, whereas fibre intake was below the daily recommended intake, and only 23% of the children achieved 100% DRI for fibre. In addition, in our analysis we adjusted for protein intake. A higher fibre intake in our results is thus coupled to a lower protein/fibre ratio and could be interpreted accordingly. Rossi et al. reported an association between protein/fibre index and serum pCS and IxS levels, while fibre intake alone was associated with pCS and not IxS, and dietary protein intake with neither of these toxins [28]. This is in line with our results, as most likely, the lack of association between protein intake and toxin concentrations is due to a low variability in protein intake, and the variability of the protein/fibre ratio is thus completely due to variation in fibre intake.

Theoretically, a diet with a lower protein/fibre ratio should be reflective of a low nitrogen/carbohydrate ratio in the colon, thus promoting carbohydrate fermentation and subsequently decreasing the production of PBUTs. Our data point to the fact that fibre intake is largely inadequate in children with CKD [25]. Some children who are exclusively fed with a powdered amino acid formula or a formula adapted to the needs of patients with CKD have even no fibre intake at all. In addition, the popular practice of protein restriction in adults with CKD stage 3–5 contrasts sharply with the minimum 100−140% DRI for proteins to maintain proper growth in children [41]. The low fibre intake in children is therefore far outweighed by the protein intake, resulting in a relatively high baseline protein/fibre ratio in comparison to adults and predisposing to proteolytic microbial activity. Future prebiotic intervention trials in children should thus aim to provide ample fibre content, while allowing sufficient protein intake and avoiding too high potassium loads. Further studies, adding the taxonomic and functional gut microbial profile to the equation, are needed to demonstrate causal relationships and unravel the meaning and relevant clinical implications of these findings.

Fibre supplementation is an appealing strategy to attenuate PBUT generation and its subsequent burden. On population level, early stages of CKD have a much higher prevalence, and an easy and relatively cheap intervention that can delay the progression of kidney disease and dialysis initiation is therefore a worthwhile intervention to further explore. Fibre intake is underappreciated and deserves more attention in the classic nutritional approach of the patient with CKD, which is largely based on sodium, phosphorous and potassium restriction. Dietary fibre is mainly supplied by the consumption of fruits and vegetables, which are often restricted to avoid hyperkalaemia. Such dietary restrictions may, however, worsen dysbiosis and further contribute to uraemic toxicity [42,43].

Limitations of the present study include the observational nature which limits causal inference and possible measurement error inherent to dietary recalls, despite careful evaluation. Transit time and microbiota composition were not assessed. Since urine samples of the children were not collected, urinary excretion of the respective uraemic toxins, as markers for generation, could not be estimated. Although we also focused on IAA and pCG, in addition to the well-known IxS and pCS, conclusions cannot be extended to the expanding array of other microbial metabolites of interest. Finally, we only included a small group of patients with CKD stage 4–5 from a single country. Accordingly, cultural and geographic effects could be at play, limiting extrapolation to other settings or populations with different dietary habits.

This study also has several strengths. To our knowledge, this is the first longitudinal study evaluating the effect of fibre intake on PBUTs in a cohort of children with non-dialysis CKD. The analysis of multiple PBUT measurements, taking into account the fluctuations within one patient over time, bridges the existence of intra-patient variability of UTs. In addition, nutrient intake was captured through repeated dietary assessments, which allowed us to account for possible intra-individual day-to-day variations as well as seasonality effects. Furthermore, we expanded our focus beyond the most thoroughly studied pCS and IxS, including pCG, which is the less concentrated glucuronidated fraction of p-cresol and IAA, the latter being, just as IxS, a product of tryptophan, originating directly in the colon by an alternative microbial metabolism pathway [44].

## 4. Conclusions

In conclusion, increasing amounts of fibre intake were associated with overall lower PBUT levels, and this independent of eGFR. As most children with CKD have a low fibre intake and a high protein requirement to allow growth, the protein/fibre ratio is re-latively high in comparison to adults, and this predisposes them to proteolytic microbial activity. The present observational findings are helpful to guide the development of well-conceived randomised controlled trials evaluating well-balanced dietary interventions in the paediatric population.

## 5. Materials and Methods

### 5.1. Study Population

For this study, longitudinal data were analysed from the multicentric, prospective, observational UToPaed study, running from 1 September 2015 to 31 December 2017. Children younger than 18 years of age with CKD stage 1–5, including transplant recipients, were eligible for inclusion. In accordance with the eGFR, determined by the updated Schwartz equation [45], CKD patients (defined using the Kidney Disease Improving Global Outcomes (KDIGO) guidelines) were stratified into stages: stage 1: ≥90 mL/min/1.73 m^2^; stage 2: 60–89 mL/min/1.73 m^2^; stage 3: 30–59 mL/min/1.73 m^2^; stage 4: 15–29 mL/min/1.73 m^2^; stage 5: <15 mL/min/1.73 m^2^. Children receiving any type of dialysis during follow-up were excluded from the analysis. Other exclusion criteria were the presence of malignancies, active infections or active systemic inflammatory disease. Participants were recruited from the Departments of Paediatric Nephrology of Ghent University Hospital, Antwerp University Hospital, University Hospitals Leuven and University Hospital Saint-Luc, Brussels. Ethical approval was granted by each participating site (number 2600/304, B670201524922; B670201422206). Prior to enrolment, written informed consent was obtained from all parents and patients above the age of 12.

### 5.2. Data Collection and Biochemical Measurements

Patients were followed prospectively during 24 months. Demographic parameters were recorded at baseline. In addition, clinical parameters (age, height, weight, etc.), dietary intake as detailed below and medical therapy were recorded at each visit. Blood samples were allowed to clot for 20–30 min and then centrifuged (2095× *g*; 10 min; 4 °C). Serum aliquots were stored at −80 °C awaiting batch analysis. Standard lab assays at the Clinical Laboratory of the Ghent University Hospital (Ghent, Belgium) were used to measure biochemical parameters including urea, creatinine (Photometric (Architect c16000, Abbott, IL, USA)), C-reactive protein, albumin and total protein. Concentrations of IxS, IAA, pCS and pCG were quantified as previously described [46]. Briefly, for total concentrations, serum samples were deproteinised by heat denaturation, followed by a filtration step through Amicon Ultra 0.5 mL filters (molecular weight cut-off 30 kDa, Millipore Merck, Darmstadt, Germany). For the free fraction, untreated plasma samples were filtered first through the Amicon Ultra Filters. Reversed-phase ultra-performance liquid chromatography (UPLC; Agilent 1290 Infinity device) (Agilent, Santa Clara, CA, USA) was used to separate the uraemic toxins. IxS (λex: 280 nm, λem: 376 nm), pCS, pCG (λex: 264 nm, λem: 290 nm) and IAA (λex: 280 nm, λem: 350 nm) were detected by an Agilent G1316C fluorescence detector.

### 5.3. Dietary Assessment

Participants’ dietary intakes were assessed 3-monthly by either a 3-day food record or a 24-h dietary recall at an aimed 50/50 ratio. Structured 3-day diary templates were completed prior to the visit and reviewed by a trained dietician in face-to-face interviews. The 3-day food record was substituted by a 24-h recall in case parents/patients failed to fill it out or forgot to bring it to the consult, so that dietary data could be coupled to serum PBUT levels from the same day. In order to increase the accuracy of portion size estimation for 24-hour recalls, standardised food models and a food photo album (Portiegroottes boek, Valetudo Consulting, third edition, march 2014) were utilised, along with a manual for the conversion of household measures to weight equivalents [47]. Fibre, protein and energy consumption were calculated by entering dietary data into Evry-Diëtist 6.7.7.0 (Evry BV, Alphen aan den Rijn, The Netherlands), based on the Belgian Branded Food Products Database (Nubel, 5th edition). A search in either the Dutch nutrient database (Nevo, 4th edition) or the online database of trade names (Internubel) was done in case of unknown food items. These were recorded in our coding book, together with standard recipes to ensure reproducibility and accuracy. Non-standard recipes and compound ingredients were broken down into their constituents. Percentage Dietary Reference Intake (%DRI) for fibre was calculated by expressing the total dietary fibre intake per patient as a percentage of the age-dependent DRI for fibre [48]. Because of the age dependency of the Belgian nutrition recommendations of protein and fibre and the small sample size for analyses in each subgroup, intakes were corrected for body surface area (BSA), calculated by the Haycock formula (0.024265 × height (cm) 0.3964 × weight (kg) 0.5378). The protein/fibre ratio was calculated.

### 5.4. Statistical Analyses

Descriptive data are expressed as mean ± standard deviation (SD) or median (25th; 75th percentile), as appropriate. Absolute and relative frequencies are reported for categorical variables.

Linear mixed-effects models were fitted to analyse the association between dietary fibre intake and the four selected gut-derived PBUTs. A compound symmetry covariance matrix was used to take into account a fixed correlation between measurements from the same patient. Linear mixed models for (natural) log-transformed plasma concentrations were fitted with a random intercept for the patient and with fibre intake (g/day), protein intake (g/day), BSA as a proxy for age (m^2^), eGFR (mL/min/1.73 m^2^). Exponentiated regression coefficients with corresponding 95% confidence intervals (CI) are reported. These reflect the geometric mean ratios. Based on the variance inflation factor, there was no indication for multicollinearity between the different explanatory variables.

All hypothesis tests were performed at the two-sided 5% significance level. Descriptive analyses were performed using SPSS 25.0 (IBM, New York, NY, USA), while the package “lme4” in R version 3.6.1 was used for linear mixed-model analyses. Statistical analyses were executed by an independent biostatistician.

## Figures and Tables

**Table 1:** Baseline characteristics of the study population (n = 61).

Variables	Values	
**Demographics**		
Age (years)	9.3 ± 5.0 (1.0–18.0)	
Gender: male	44 (72)	
Transplant recipients	8 (13)	
**Anthropometry**		
Weight SDS	−1.0 ± 1.4	
Height SDS	−1.2 ± 1.2	
BMI SDS	−0.3 ± 1.3	
BSA (m^2^)	1.0 ± 0.4	
**Cause of kidney failure**		
Glomerular	11 (18)	
CAKUT	27 (44)	
Cystic disease	6 (10)	
Other non-glomerular	17 (28)	
**Laboratory values**		
eGFR (ml/min/1.73 m^2^)	47.1 ± 28.9	
**Chronic medication use**		
Potassium binding resins	7 (12)	
Phosphate binders	2 (3)	
Iron supplements	22 (36)	
Immunosuppressive therapy	10 (16)	
Laxatives	1 (3)	
Antibiotics	19 (31)	
**Nutrient intake**		
Fibre intake (g/day/m^2^)	12.6 ± 6.8	
%DRI fibre	76.0 ± 36.0	
Protein intake (g/day/m^2^)	54.4 ± 28.4	
%DRI protein	220.1 ± 138.2	
Protein/fibre index	4.3 (2.7–5.8) *	
Energy (kCal/kg/day)	59.8 ± 30.9	
Energy (kCal/day)	1428.4 ± 504.9	
**Gut-derived protein-bound uraemic toxins**		
	**Free**	**Total**
pCG (mg/dL)	0.004 (0.001–0.013)	0.006 (0.001–0.015)
IAA (mg/dL)	0.004 (0.002–0.007)	0.042 (0.028–0.061)
IxS (mg/dL)	0.006 (0.003–0.015)	0.247 (0.101–0.514)
pCS (mg/dL)	0.017 (0.006–0.034)	0.761 (0.269–1.372)

CAKUT: congenital anomalies of the kidney and urinary tract; SDS: standard deviation score; BMI: body mass index; BSA: body surface area; eGFR: estimated glomerular filtration rate according to Schwartz et al.; %DRI: achieved percentage of the recommended 100% dietary reference intake; pCG: p-cresylglucuronide; IAA: indole acetic acid; IxS: indoxyl sulfate; pCS: p-cresyl sulfate. Data are expressed as mean ± standard deviation (SD), number (percentage) or median (25th–75th percentile) as appropriate. * n = 58, three patients with a fibre intake of 0 g/day were excluded for mathematical reasons.

**Table 2:** Association between nutrient (fibre and protein) intake and selected gut-derived protein-bound uraemic toxins.

Gut-Derived Protein-Bound Uraemic Toxins (mg/dL)	Estimated Mean Ratio	Confidence Interval (CI)		*p*
		Lower 95%	Upper 95%	
**pCG**				
Free concentration				
Fibre intake (g/day)	0.967	0.943	0.992	0.010
Protein intake (g/day)	1.005	0.998	1.012	0.120
Total concentration				
Fibre intake (g/day)	0.970	0.944	0.995	0.021
Protein intake (g/day)	1.006	0.998	1.012	0.116
**IAA**				
Free concentration				
Fibre intake (g/day)	0.934	0.907	0.963	<0.001
Protein intake (g/day)	1.011	1.003	1.019	0.007
Total concentration				
Fibre intake (g/day)	0.984	0.971	0.997	0.020
Protein intake (g/day)	1.001	0.998	1.005	0.430
**IxS**				
Free concentration				
Fibre intake (g/day)	0.969	0.941	0.997	0.035
Protein intake (g/day)	1.005	0.997	1.012	0.259
Total concentration				
Fibre intake (g/day)	0.986	0.965	1.006	0.196
Protein intake (g/day)	1.001	0.996	1.007	0.645
**pCS**				
Free concentration				
Fibre intake (g/day)	0.975	0.953	0.998	0.034
Protein intake (g/day)	1.005	0.998	1.011	0.137
Total concentration				
Fibre intake (g/day)	0.984	0.956	1.011	0.261
Protein intake (g/day)	1.004	0.9962	1.012	0.282

Total and free uraemic toxin concentrations showed a skewed distribution and were (natural) log-transformed prior to linear mixed-model analysis. Data are expressed as estimated mean ratio and 95% confidence interval (CI).

## Data Availability

The datasets generated during and/or analysed during the current study are available from the corresponding author on reasonable request.

## Supplementary Materials

The following are available online at https://www.mdpi.com/2072-6651/13/3/225/s1, Table S1: Nutrient intake across different CKD stages, Table S2: Serum concentrations of total and free gut-derived, protein-bound uraemic toxins across different CKD stages.
