# Supplementary material for: Dietary Fibre Intake Is Associated with Serum Levels of Uraemic Toxins in Children with Chronic Kidney Disease
Source: Toxins (Basel). 2021 Mar 19;13(3):225. doi: 10.3390/toxins13030225 (PMC8003569; doi:10.3390/toxins13030225)
Supplement: Supplementary file 1 [file toxins-13-00225-s001.pdf]

Supplementary Materials: Dietary Fibre Intake is Associated with Serum Levels of Uraemic Toxins in Children with Chronic Kidney Disease

Amina El Amouri, Evelien Snauwaert, Aurélie Foulon, Charlotte Vande Moortel, Maria Van Dyck, Koen Van Hoeck, Nathalie Godefroid, Griet Glorieux, Wim Van Biesen, Johan Vande Walle, Ann Raes and Sunny Eloot

Table S1. Nutrient intake of patients across different CKD stages.

Nutrient intake	CKD 1–2 (n = 20)	CKD 3 (n = 19)	CKD 4–5 (n = 22)	*p*
Fibre intake (g/day/m²)	15.8 ± 6.7	12.5 ± 6.9	9.9 ± 5.8	0.017°
% DRI fibre	91.7 ± 35.3	78.0 ± 32.3	60.0 ± 34.1	0.014°°
Protein intake (g/day/m²)	64.8 ± 39.7	52.9 ± 19.2	46.4 ± 19.7	0.104
%DRI protein	274 ± 196	202 ± 90	187 ± 93	0.101
Protein/Fibre index	4.2 (3.2–4.7)	5.0 (2.7–6.2)	3.8 (2.8–6.1)*	0.552
Energy (kCal/day)	1505 ± 478	1463 ± 491	1329 ± 546	0.503
Energy (kCal/kg/day)	68.2 ± 35.4	50.5 ± 27.8	60.1 ± 27.9	0.204

%DRI: achieved percentage of the recommended 100% Dietary Reference Intake. Data are expressed as mean ± standard deviation (SD) or median (25th–75th percentile) as appropriate. *n = 19, the three patients with a fibre intake of 0 g/day were excluded for mathematical reasons. ° Significantly higher fibre intake in CKD 1–2 patients versus CKD 4–5 patients (6 g/day/m², 95%CI: 1–11 g, $p = 0.012$). °°Significantly higher %DRI for fibre intake in CKD 1-2 patients versus CKD 4-5 patients (32%, 95%CI: 7–57%, $p = 0.010$). Comparison between the CKD stages was done using a one-way ANOVA test with post-hoc Tukey comparison or a Kruskal–Wallis test with Bonferroni correction as appropriate.

Table S2. Serum concentrations of total and free gut-derived, protein-bound uraemic toxins in patients across different CKD stages.

Gut-derived, protein-bound uraemic toxins (mg/dL)		CKD 1–2 (n = 20)	CKD 3 (n = 19)	CKD 4–5 (n = 22)
**pCG**				
	Total	0.001 (0.001–0.009)	0.001 (0.001–0.008)	0.017 (0.007–0.043)
	Free	0.001 (0.001–0.006)	0.001 (0.001–0.007)	0.015 (0.006–0.039)
**IAA**				
	Total	0.032 (0.019–0.050)	0.036 (0.028–0.051)	0.062 (0.036–0.078)
	Free	0.002 (0.001–0.003)	0.003 (0.002–0.006)	0.008 (0.004–0.110)
**IxS**				
	Total	0.082 (0.047–0.168)	0.223 (0.102–0.264)	0.573 (0.458–0.739)
	Free	0.003 (0.001–0.005)	0.004 (0.001–0.007)	0.019 (0.011–0.031)
**pCS**				
	Total	0.250 (0.073–0.835)	0.665 (0.352–0.860)	1.569 (0.973–2.159)
	Free	0.008 (0.003–0.017)	0.012 (0.006–0.021)	0.043 (0.018–0.074)

pCG: p-cresylglucuronide; IAA: indole acetic acid; IxS: indoxyl sulfate; pCS: p-cresyl sulfate. Data are expressed as median (25th–75th percentile).
